# Structures of NS5 Methyltransferase from Zika Virus

**DOI:** 10.1016/j.celrep.2016.08.091

**Published:** 2016-09-12

**Authors:** Javier Coloma, Rinku Jain, Kanagalaghatta R. Rajashankar, Adolfo García-Sastre, Aneel K. Aggarwal

**Affiliations:** 1 Department of Pharmacological Sciences, Icahn School of Medicine at Mount Sinai, New York, NY 10029, USA; 2 Department of Chemistry and Chemical Biology, Cornell University, Ithaca, NY 14853, USA; 3 NE-CAT, Advanced Photon Source, Argonne, IL 60439, USA; 4 Department of Microbiology, Icahn School of Medicine at Mount Sinai, New York, NY 10029, USA; 5 Global Health and Emerging Pathogens Institute, Icahn School of Medicine at Mount Sinai, New York, NY 10029, USA; 6 Department of Medicine, Division of Infectious Diseases, Icahn School of Medicine at Mount Sinai, New York, NY 10029, USA

## Abstract

The Zika virus (ZIKV) poses a major public health emergency. To aid in the development of antivirals, we present two high-resolution crystal structures of the ZIKV NS5 methyltransferase: one bound to S-adenosylmethionine (SAM) and the other bound to SAM and 7-methyl guanosine diphosphate (7-MeGpp). We identify features of ZIKV NS5 methyltransferase that lend to structure-based antiviral drug discovery. Specifically, SAM analogs with functionalities on the Cβ atom of the methionine portion of the molecules that occupy the RNA binding tunnel may provide better specificity relative to human RNA methyltransferases.

## In Brief

Zika virus (ZIKV) has emerged as a major health concern. To aid the development of antivirals, Coloma et al. report high-resolution crystal structures of the ZIKV NS5 methyltransferase, one bound to S-adenosylmethionine and another bound to S-adenosylmethionine and 7-methyl guanosine diphosphate. The structures provide a basis for antiviral drug discovery.

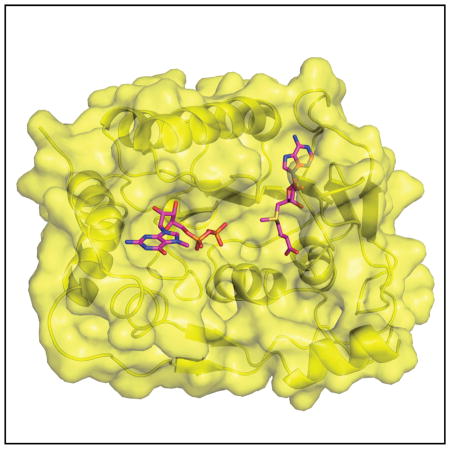

## INTRODUCTION

The Zika virus (ZIKV) is a member of the *Flavivirus* genus that includes other mosquito-borne human pathogens such as dengue virus (DENV1–4), Murray Valley encephalitis virus (MVEV), West Nile virus (WNV), yellow fever virus (YFV), and Japanese encephalitis virus (JEV), among others (Petersen et al., 2016). ZIKV has emerged as a major health concern over the past year (Fauci and Morens, 2016; Lazear and Diamond, 2016). Its rapid spread across the Americas and, in particular, its link to microcephaly in newborn infants and the Guillain-Barré syndrome in adults has invigorated efforts to develop a vaccine against ZIKV and to eradicate the *Aedes* mosquito vectors. An important strategy for halting the spread of ZIKV in the event of more outbreaks would be designing antivirals to inhibit viral protein enzymatic activities central to the lifecycle and survival of the virus.

The flavivirus genome consists of an ~11 kB positive-sense single-stranded RNA that acquires a methylated 5′ cap structure (^N7Me^GpppA_2′OMe_; Me, methyl group) for stability, efficient translation, and evasion of the host immune response (Dong et al., 2014). Both the N7 and 2′O methylation reactions are performed by the same methyltransferase (MTase) domain, located at the N terminus of the nonstructural protein NS5. The NS5-MTase methylates first the N7 atom of guanosine and then the 2′O of the initiating adenosine of the nascent viral transcript (GpppA-RNA →^N7Me^GpppA-RNA→^N7Me^GpppA_2_′_OMe_-RNA) using S-adenosylmethionine (SAM) as the methyl donor and generating S-adenosylhomocysteine (SAH) as the reaction byproduct (Dong et al., 2014). Crystal structures of several flavivirus NS5-MTases have been reported bound to various ligands (Bollati et al., 2009; Egloff et al., 2002, 2007; Yap et al., 2010; Zhao et al., 2015; Zhou et al., 2007), including SAM/SAH, GTP, and RNA.

Mutations in NS5-MTase that lead to defects in N7 methylation are lethal in flaviviruses (Dong et al., 2010; Ray et al., 2006; Zhou et al., 2007), whereas defects in 2′O methylation attenuate the virus and is a basis for vaccine development (Li et al., 2013; Züst et al., 2013). NS5-MTase has become an attractive target for the development of antivirals to block cap formation, and several inhibitors have been reported bound to either the SAM/SAH binding pocket or the GTP binding pocket (Benarroch et al., 2004; Chen et al., 2013; Coutard et al., 2014; Lim et al., 2011; Stahla-Beek et al., 2012). To help guide the discovery of antivirals against ZIKV, we present two high-resolution crystal structures of NS5-MTase from the French Polynesia strain of the ZIKV virus. The first structure, determined at 1.33 Å resolution, has SAM bound to the enzyme (NS5-MTase_SAM_). The second structure, determined at 1.50 Å resolution, has both SAM and N7-methyl guanosine diphosphate (7-MeGpp) bound to the enzyme (NS5-MTase_SAM,7-MeGpp_). The high resolution of both structures makes them well suited for structure-based antiviral drug discovery.

## RESULTS

**Figure 1:**
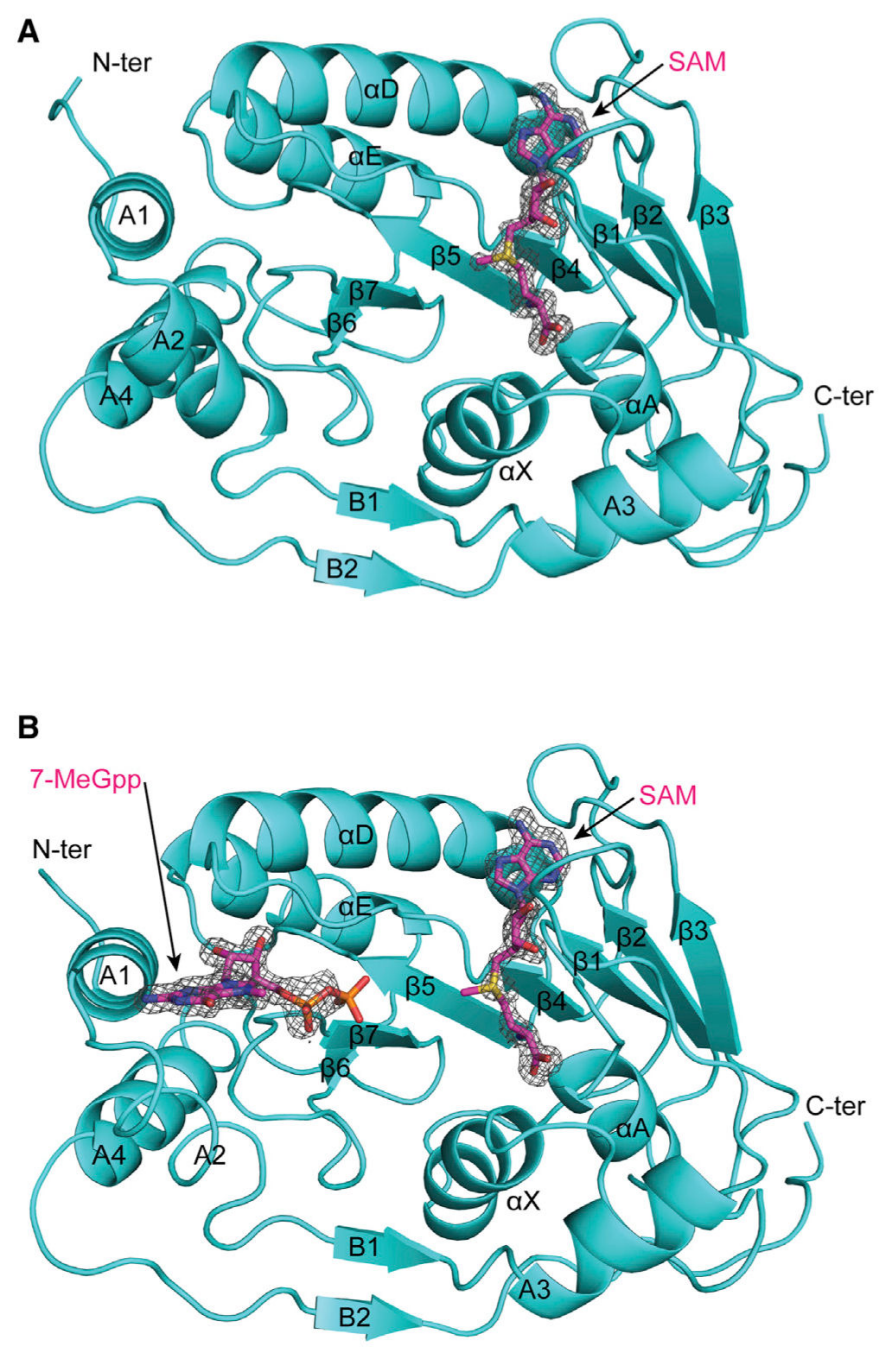
Structures of ZIKV NS5-MTase (A) Structure of ZIKV NS5-MTase bound to SAM. Fo-Fc difference electron density (contoured at 3σ) for SAM is shown as gray mesh. Secondary structure elements are labeled. Ter, terminus. (B) Structure of ZIKV NS5-MTase bound to SAM and 7-MeGpp. Fo-Fc difference electron density (contoured at 3σ) for SAM and 7MeGpp is shown as gray mesh.

We expressed and purified ZIKV NS5-MTase (residues 1–266) of the H/PF/2013 strain as a soluble protein from *E. coli*. Purified protein was crystallized at 20°C, and the structure was determined by molecular replacement. Initial maps revealed difference density in the SAM binding pocket (Figure 1A) despite no intentional addition of the ligand in the purification or crystallization buffers. It is common for flavivirus MTases to copurify with SAM or SAH as, for example, in structures of NS5-MTase from DENV2 (PDB: 1L9K) (Egloff et al., 2002) and DENV3 (PDB: 3P97) (Lim et al., 2011). The structure of NS5-MTase_SAM_ was refined to a resolution of 1.33 Å, with R_work_ and R_free_ values of 17.0% and 18.9%, respectively (Table 1). To obtain crystals of NS5-MTase_SAM,7-MeGpp_, SAM and 7-MeGpp were added to purified ZIKV NS5-MTase in 6-fold molar excess prior to crystallization. The structure of NS5-MTase_SAM,7-MeGpp_ was determined to 1.50 Å with R_work_ and R_free_ values of 15.9% and 17.9%, respectively (Table 1).

**Figure 2:**
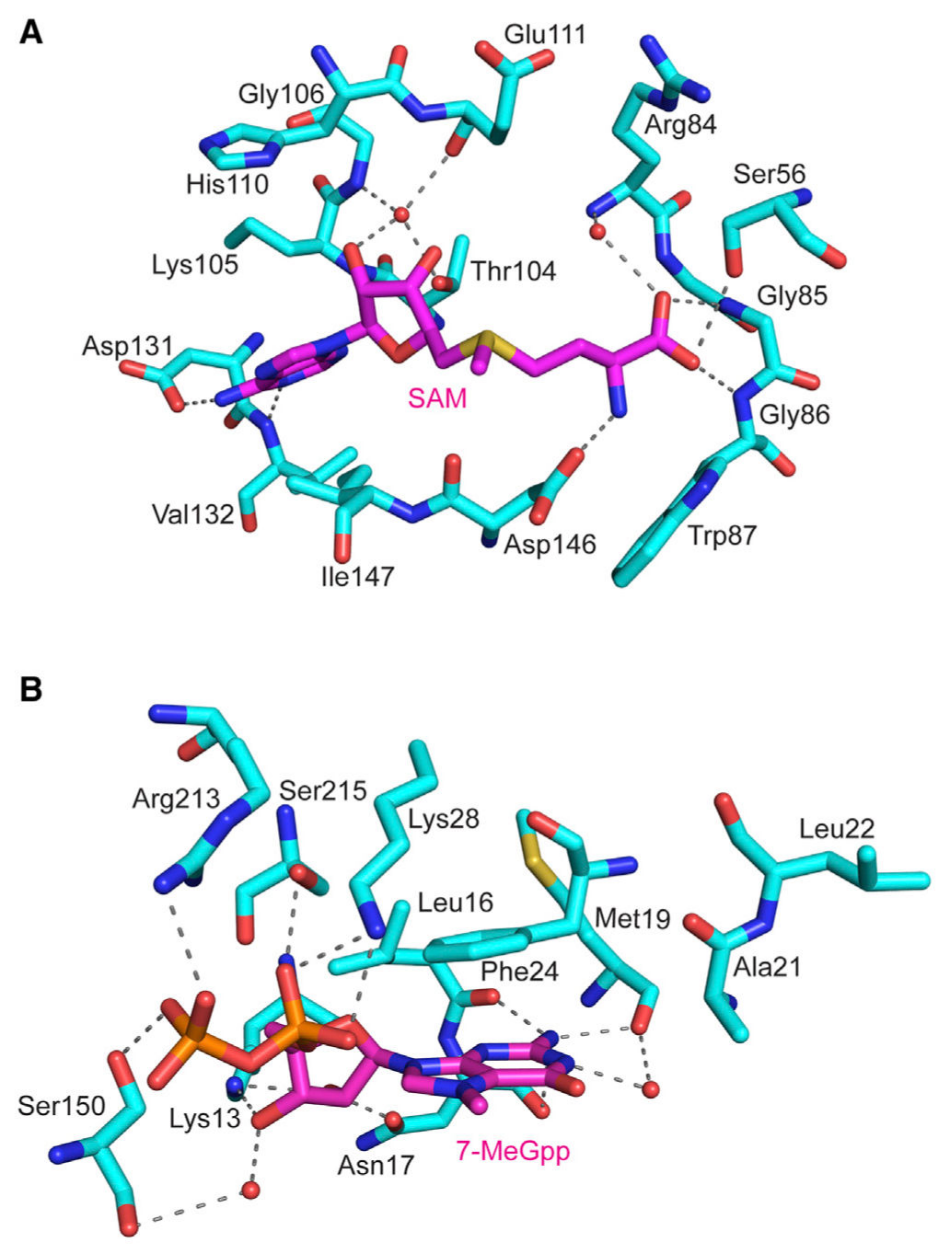
Interactions of ZIKV NS5-MTase with Bound Ligands (A) Interactions between ZIKV NS5-MTase and SAM. Hydrogen bonds are depicted as dashed lines. Select solvent molecules are shown as red spheres. (B) Interactions between ZIKV NS5-MTase and 7-MeGpp.

The two structures are nearly identical and superimpose with an RMSD of 0.21 Å for 219 Cα atoms, revealing an enzyme that is essentially preformed to bind 7-MeGpp (Figure 1). The ZIKV NS5-MTase consists of a MTase core (residues 54–223) with a Rossmann fold, composed of seven β strands (β1–β7) and four α helices (αX, αA, αD, and αE) (Figures 1 and S1). The MTase core is flanked by α helices (A1–A4) and β strands (B1–B2) that form the N- and C-terminal extensions and cradle one side of the core (Figure 1). SAM binds in a cleft formed by β1, β2, and αA as well as a loop (amino acids 81–86) that carries the consensus “motif 1” (GxGxGx; x, usually a small chain amino acid) characteristic of Rossmann fold methylases (Figures 1A and 2A). The SAM adenine base is sandwiched between the Lys105 (partially disordered) and Ile147 side chains, whereas the N1 and N6 atoms makes hydrogen bond with the backbone amide of Val132 and side chain of Asp131 (Figure 2A). The ribose sugar participates in solvent mediated interactions with the backbone of Gly106 and Glu111 and side chain of Thr104. The methionine portion of SAM is fixed by interactions with the side chains of Ser56 and Asp146 as well as the backbone amides of Gly86 and Trp87. Asp146 is part of the K_61_-D_146_-K_182_-E_218_ tetrad motif that has been shown to be important for both N7 and 2′O methylation. 7-MeGpp binds in a cleft between the helices A1, A2, and a loop between β6 and β7 (amino acids 208–218). The 7-MeGpp base packs against the aromatic ring of Phe24, whereas its exocyclic N6 amino group makes hydrogen bonds with the main chain carbonyls of Leu16 and Met19 (Figures 1B and 2B). The diphosphate moiety is fixed by hydrogen bonds with Lys28, Ser150, Arg213, and Ser215, whereas the sugar 2′O is within hydrogen bonding distance of the side chains of Lys13 and Asn17. All of these amino acids are conserved in flaviviruses and underlie almost identical interactions with bound ligands (Figure S1).

**Figure 3:**
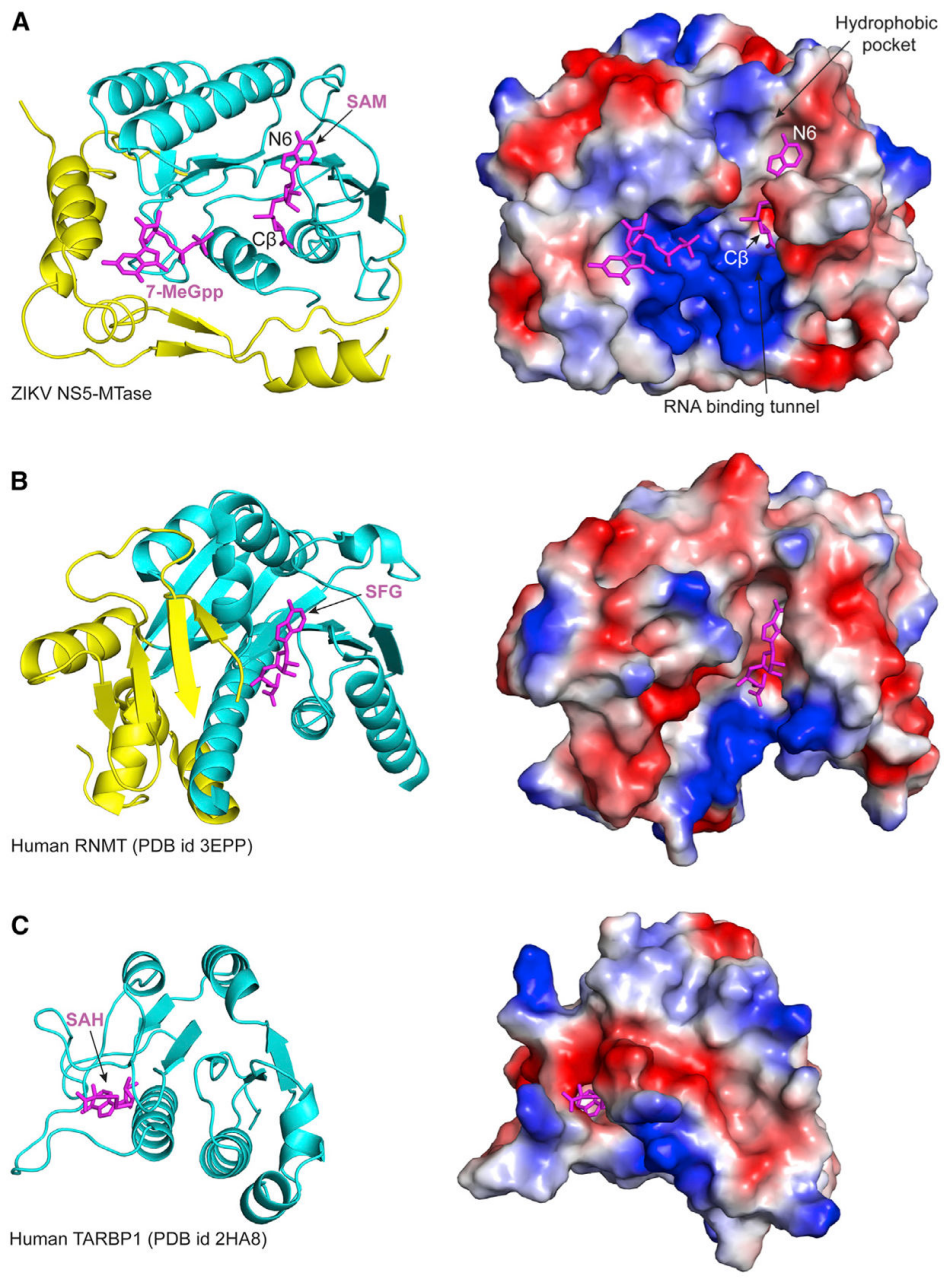
Comparison between ZIKV NS5-MTase and Human RNA MTases (A) ZIKV NS5-MTase shown with secondary structure (left) and electrostatic surface (right). The secondary structure elements corresponding to the MTase core is colored cyan, whereas the secondary structure elements from N- and C-termini are colored yellow. Blue and red colors on the electrostatic surface indicate positive and negative electrostatic potential, respectively. SAM and 7-MeGpp are shown in magenta. The hydrophobic cavity adjacent to the N6 atom on adenine of SAM is labeled (Lim et al., 2011). The RNA binding tunnel adjacent to the methionine portion (and atom Cβ) of SAM is also labeled. (B) Human mRNA cap guanine-N7 methyltransferase (RNMT; PDB: 3EPP) shown with secondary structure (left) and electrostatic surface (right). The protein is aligned to the central β sheet of the ZIKV NS5-MTase core. Sinefungin (SFG) is shown in magenta. (C) The MTase domain of human TAR (HIV-1) RNA binding protein 1 (TARBP1; PDB: 2HA8) (Wu et al., 2008). The protein is aligned to the central β sheet of the ZIKV NS5-MTase core. S-adenosylhomocysteine (SAH) is shown in magenta.

The SAM and 7-MeGpp binding sites are separated by >15 Å (Figure 3A). A positively charged tunnel spans two sites where, based on the structure of DENV3 NS5 with RNA (Zhao et al., 2015), the ZIKV viral RNA likely binds (Figure 3A) and interacts with residues Lys28, Arg41, Arg57, Arg84, Glu111, and Arg213 (Figure S1). Interestingly, there is significant conformational variability in these residues in the various flavivirus structures, reflecting the absence of RNA in most of these structures. The ZIKV NS5-MTase structures superimpose very well on MTase structures from other flaviviruses (Figure S2). Most of the main-chain variability is in three solvent exposed loops (residues 48–52, 173–178, and 244–251) that diverge the most in sequence (Figures S1 and S2). Curiously, the ZIKV MTase contains Ala21 and Leu22 near the 7-MeGpp binding site (Figure S1) instead of Arg/Lys and Glu/Arg/Lys/Ser in other flaviviruses, which lends to a slightly more hydrophobic GTP binding pocket. Ala21 and Leu22 are conserved in every strain of ZIKV that has been examined thus far. The ZIKV MTase is also notable in containing Thr188 and Ser189 instead of Met and Pro in other flaviviruses, which manifests in a small shift in helix αE (Figure S2).

## DISCUSSION

Here, we present two high-resolution crystal structures of ZIKV NS5-MTase: one with SAM bound to the enzyme, and the other with both SAM and 7-MeGpp bound to the enzyme as a mimic of the substrate complex (without RNA) for the ^N7Me^GpppA-RNA→^N7Me^GpppA_2′OMe_-RNA step of the reaction. Because of the necessity of N7 methylation, the ZIKV NS5-MTase presents an attractive target for the development of inhibitors. One challenge is the design of inhibitors that are specific for a flavivirus MTase without affecting the host RNA MTases, such as the human mRNA cap guanine-N7 methyltransferase (RNMT; PDB: 3EPP) and the MTase domain of human TAR (HIV-1) RNA binding protein 1 (TARBP1; PDB: 2HA8) (Wu et al., 2008) (Figures 3B and 3C). A step in that direction is a reported SAH analog with a N6 substituent (a benzyl ring) that fits into a hydrophobic cavity adjacent to the SAM binding pocket (Lim et al., 2011) (Figure 3A). This hydrophobic cavity is conserved in flavivirus MTases (Lim et al., 2011) (including ZIKV) but is not found in human N7/2′O MTases (Figure 3). From the structures, another approach is to develop SAM/SAH analogs with functionalities that access the positively charged RNA binding tunnel (Figure 3A). The tunnel extends from the SAM binding pocket and substituents on the Cβ atom of the methionine portion of SAM/SAH would be well positioned to enter the RNA binding tunnel and interact with flavivirus specific residues (Figure 3A). The emerging role of histone methyltransferases in cancer has spurred the development of new SAM competitors (Kaniskan and Jin, 2015; Luo, 2012), including some SAM/SAH analogs (Daigle et al., 2011). Some of these compounds have entered clinical trials for various cancer malignancies; it will be interesting to see whether any of these compounds can also inhibit the ZIKV MTase.

In conclusion, the high-resolution structures of ZIKV NS5-MTases presented here provide a basis for structure-based antiviral drug discovery. The SAM and GTP binding pockets and the RNA binding tunnel present hotspots for in silico and fragment based screening. From the structures, SAM/SAH analogs with functionalities on the Cβ atom of the methionine portion of the molecules can potentially co-occupy the RNA binding tunnel and provide better specificity against human RNA MTases.

## EXPERIMENTAL PROCEDURES

ZIKV NS5-MTase (1–266) from the H/PF/2013 strain was expressed and purified from *E. coli* strain LOBSTR (DE3) with an N-terminal His_6_-SUMO tag. Cell pellets containing the recombinant protein were resuspended in buffer containing 50% B-PER (Thermo Scientific), 25 mM Tris pH 8.0, 500 mM NaCl, 5% glycerol, and 5 mM 2-mercaptoethanol (BME). Cells were lysed by sonication and the filtered lysate was loaded on a 5 ml Ni-NTA column (QIAGEN). Protein bound to the Ni-NTA column was eluted with buffer containing 50 mM Tris-HCl, pH 8.0, 500 mM NaCl, 5% glycerol, 5 mM BME and 250 mM imidazole. Eluted protein was dialyzed into buffer containing 50 mM HEPES (pH 7.5), 500 mM NaCl, 5% glycerol, and 5 mM BME. The His_6_-SUMO tag was cleaved with Ulp protease and the protein re-loaded on the Ni-NTA column to remove the cleaved His_6_-SUMO tag and any uncleaved protein. The cleaved protein was further purified by ion exchange chromatography on an anion exchange column and by size exclusion chromatography on a SD75 column (GE Healthcare). Before crystallization, the protein was concentrated to 10 mg/mL in buffer containing 25 mM HEPES (pH 7.5), 400 mM NaCl, 5% glycerol, and 2 mM TCEP.

The NS5-MTase_SAM_ crystals grew out of drops containing 17%–20% PEG 400 and 0.1 M Tris (pH 8.0). For data collection, crystals were cryoprotected by quick dipping in mother liquor containing a mixture of 9% sucrose, 2% glucose, 8% glycerol, and 8% ethylene glycol and flash-cooled in liquid nitrogen. Diffraction data were collected at the Advanced Photon Source (beamline 24-ID-C). The data were indexed with HKL2000 (Otwinowski and Minor, 1997), and the structure was solved by molecular replacement with Auto-Rickshaw (http://webapps.embl-hamburg.de/cgi-bin/Auto-Rick/arinitAR1.cgi) (Panjikar et al., 2005). The model obtained from the Auto-Rickshaw pipeline was improved by iterative manual building and refinement with Coot (Emsley and Cowtan, 2004) and Phenix (Adams et al., 2010), respectively. After the protein chain was built, significant difference electron density (3σ) was visible in the SAH/SAM binding site (Figure 1A). The electron density was consistent with the presence of SAH/SAM despite no intentional addition of the ligand in the purification or crystallization buffers. This difference density was modeled as SAM, but the slightly lower occupancy of the methyl group (~75% occupancy) is suggestive of predominantly SAM with a small amount of SAH in the crystallized protein. The structure of NS5-MTase_SAM_ was refined to 1.33 Å (Rfree of 18.9% and Rwork of 17.0%) and contains one protein chain, one SAM molecule, one phosphate ion, one chlorine atom, and 299 solvent molecules. The model has excellent stereochemistry, as shown by MolProbity (Davis et al., 2007), with 98.1% of all residues in allowed regions of the Ramachandran plot and no residues in the disallowed regions.

To obtain crystals of The NS5-MTase_SAM,7-MeGpp_, purified protein at 0.5 mM concentration was mixed with 3 mM SAM and 3 mM 7-MeGpp prior to crystallization. Crystals used for data collection grew out of drops containing 6%–9% PEG 8K, 0.07 M sodium acetate (pH 5.0), and 30% glycerol. For data collection, crystals were flash-cooled in liquid nitrogen directly from the drops. Diffraction data were collected at the Advanced Photon Source (beamline 23-ID-D). The data were indexed with iMOSFLM (Battye et al., 2011), and the structure was solved by molecular replacement using Auto-Rickshaw. The model obtained from Auto-Rickshaw was improved by iterative manual building and refinement with Coot and Phenix, respectively. Clear electron density was visible for both SAM and 7-MeGpp (Figure 1B). Although the density for the base and sugar of 7-MeGpp was well defined, the density for the β-phosphate was not as clear. Based on the shape of the density, we modeled the β-phosphate in two conformations with 70% and 30% occupancies (Figure S3). The structure of NS5-MTase_SAM,7-MeG_ was refined to 1.5 Å (Rfree of 17.9% and Rwork of 15.9%) and contains one protein chain, one SAM molecule, one 7-MeGpp molecule, four glycerol molecules, one phosphate ion, one acetate ion, and 244 solvent molecules. The stereochemistry of the protein is excellent, with 98.1% of the residues in the most favored regions of the Ramachandran plot and none in the disallowed regions.

PyMol (https://www.pymol.org/) was used to compute qualitative surface electrostatic potential and prepare figures. The potential range was set the same for all the structures; positive potential is shown in blue, and negative potential is shown in red.

## Supplementary Material

1

2

## Figures and Tables

**Table 1:** Data Collection and Refinement Statistics

	ZIKV NS5-MTase_SAM_ (PDB: 5KQR)	ZIKV NS5-MTase_SAM,7-MeGpp_ (PDB: 5KQS)
Data Collection		
Space group	C 1 2 1	C 1 2 1
Cell Dimensions		
a, b, c (Å)	72.65, 78.14, 45.38	129.34, 77.48, 37.03
α, β, γ (°)	90.00, 106.99, 90.00	90.00, 104.40, 90.00
Resolution (Å)	50.00–1.33 (1.35–1.33)^a^	65.90–1.50 (1.53–1.50)^a^
R_sym_	0.063 (0.280)	0.053 (0.478)
I/σ(I)	17.7 (4.1)	7.7 (1.4)
Completeness (%)	91.9 (55.5)	99.0 (99.0)
Redundancy	4.7 (3.5)	1.8 (1.7)
Refinement		
Resolution (Å)	43.40–1.33	36.80–1.50
No. reflections	50670	56046
R_work_/R_free_	0.170/0.189	0.159/0.179
No. atoms		
Protein	2055	2043
Ligand/ion (SAM/7-MeGpp/others)	27/–/6	27/29/33
Water	299	244
B factors (Å^2^)		
Protein	18.6	28.5
Ligand/ion (SAM/7-MeGpp/others)	17.8/–/29.3	21.9/27.5/58.4
Water	30.6	44.1
RMSDs		
Bond lengths (Å)	0.008	0.005
Bond angles (°)	1.031	0.86

a Values in parentheses are for the highest-resolution shell.

## Highlights

- Structure of the ZIKV NS5 methyltransferase bound to SAM at 1.33 Å
- Structure of the ZIKV NS5 methyltransferase bound to SAM and 7-MeGpp at 1.5 Å
- Identification of features that lend to structure-based drug discovery
