# Supplementary material for: Structures of NS5 Methyltransferase from Zika Virus
Source: Cell Rep. Author manuscript; Available in PMC 2016 Oct 21. (PMC5074680; doi:10.1016/j.celrep.2016.08.091)
Supplement: 1 [file NIHMS814537-supplement-1.pdf]

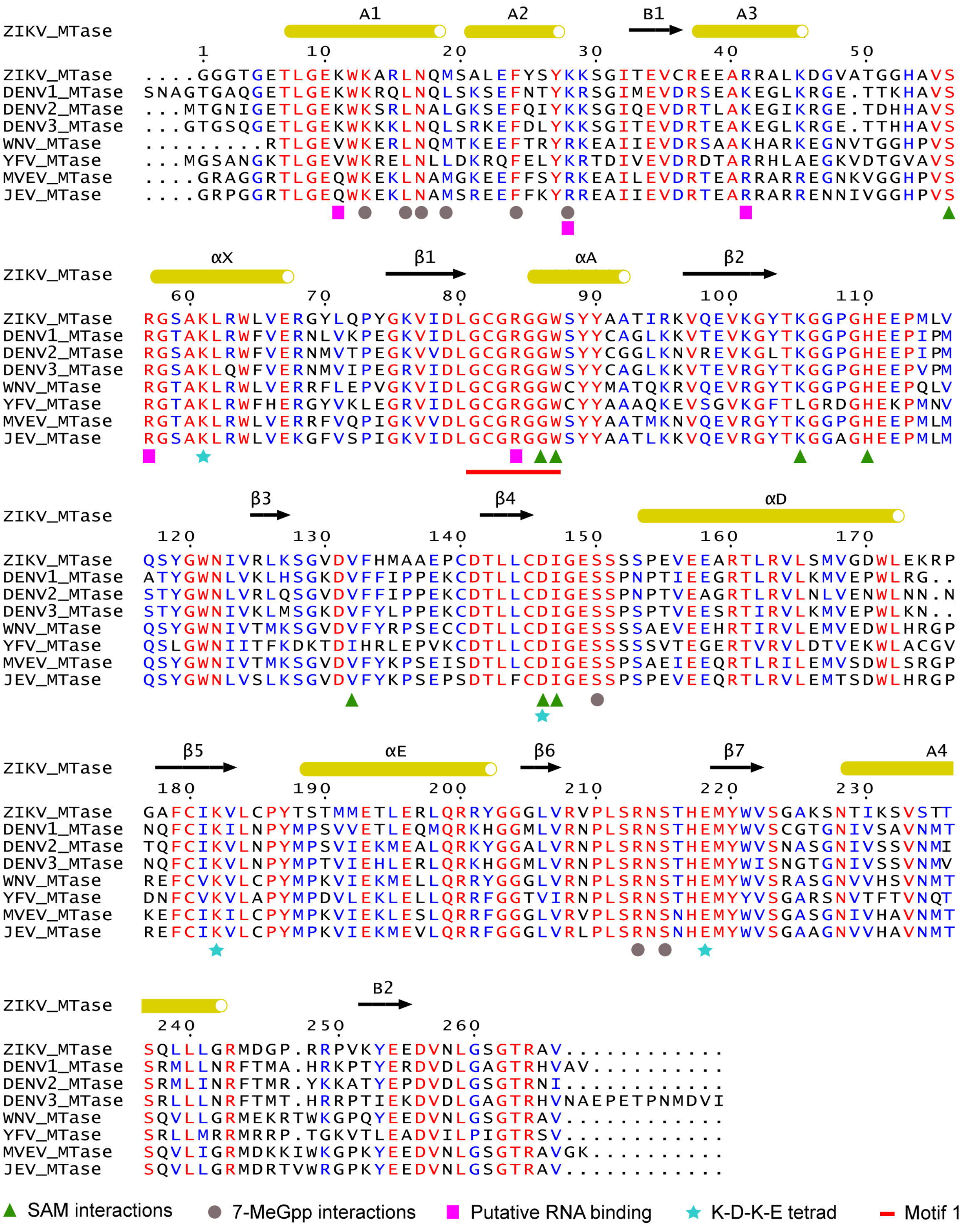

Figure S1. Sequence alignment of Flavivirus NS5-MTases. Related to Figure 1

Multiple sequence alignment of ZIKV NS5-MTase orthologs from DENV1-3, WNV, YFV, MVEV and JEV. Secondary structures of the core Rossmann fold are labeled αX, αA, αD, αE (alpha helices) and β1-β7 (beta strands). The flanking secondary structure

elements are labeled A1, A2, A3 for alpha helices and B1-B2 for beta strands. Identical and conserved amino acids are shown in red and blue respectively. SAM interacting residues are indicated with green triangles and those that interact with and 7-MeGpp are indicated with gray circles. Residues of the conserved K-D-K-E tetrad motif are indicated with cyan stars; the GxGxGx motif 1 is underlined with a red line.

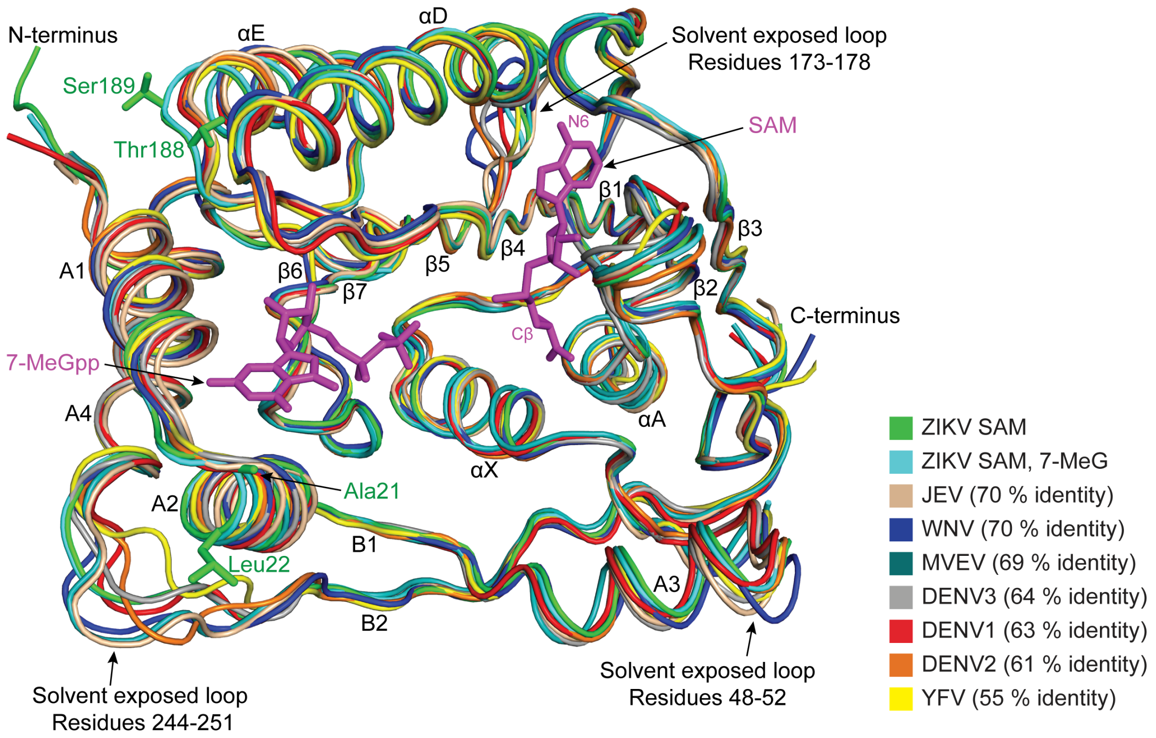

Figure S2. Comparison of flavivirus NS5-MTase domains. Related to Figure 1

PDB IDs: Japanese encephalitis virus (JEV) PDB 4K6M, West Nile virus (WNV) PDB 2Oy0, Murray Valley encephalitis virus (MVEV) PDB 2PX2, dengue virus type 3 (DENV3) PDB 3P97, dengue virus type 1 (DENV1) PDB 5IKM, dengue virus type 2 (DENV2) PDB 3EVG, and yellow fever virus (YFV) PDB 3EVA.

Superimposition of flavivirus NS5-MTase domains. S-adenosylmethionine (SAM) and 7-methylguanosine-diphosphate (7-MeGpp) bound by ZIKV NS5-MTase are shown in magenta stick; residues Ala21-Leu22 (R/K and E/R/K/S in others) and Thr188-Ser189 (Met-Pro in others) are shown in green stick. Solvent exposed loops with variable conformation are labeled.

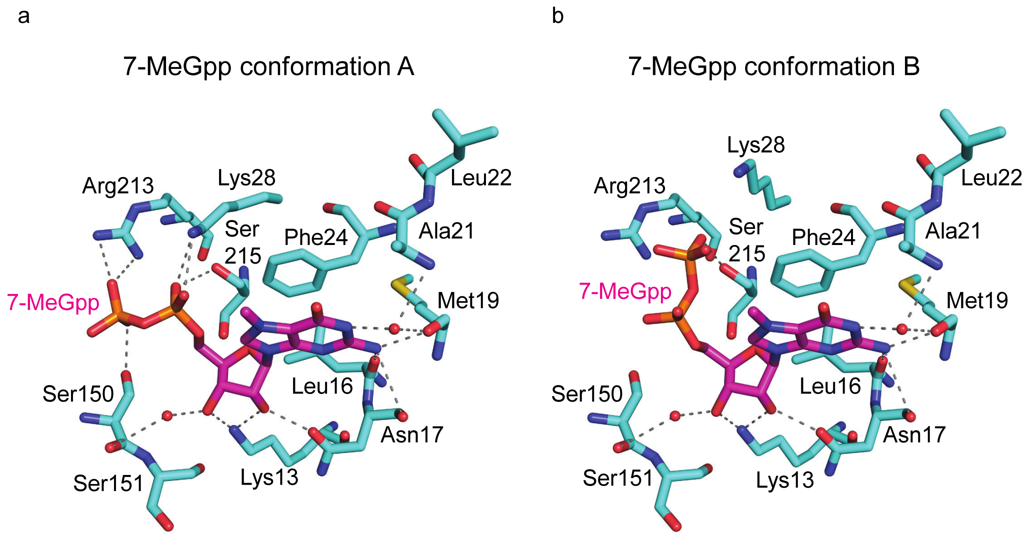

Figure S3. Dual conformation of 7-MeGpp. Related to Figure 2 and Experimental Procedures

The density for the β-phosphate of 7-MeGpp is not as well defined as the rest of the molecule, and is best modeled in two conformations: 70 % occupancy in conformation A (left) and 30 % occupancy in conformation B (right). The two conformations differ slightly in their interactions with the residues in the 7-MeGpp binding pocket.
