# Supplementary material for: Structures of NS5 Methyltransferase from Zika Virus
Source: Cell Rep. Author manuscript; Available in PMC 2016 Oct 21. (PMC5074680; doi:10.1016/j.celrep.2016.08.091)
Supplement: 2 [file NIHMS814537-supplement-2.docx]

Figure S1: Sequence alignment of flavivirus NS5-MTases, Related to Results

Figure S2: Comparison of flavivirus NS5-MTase domains, Related to Results

Figure S3: Dual conformation of 7-MeGpp, Related to Experimental Procedures
